# How Specific Is Site‐Specific? A Review and Guidance for Selecting and Evaluating Approaches for Deriving Local Water Quality Benchmarks

**DOI:** 10.1002/ieam.4181

**Published:** 2019-08-28

**Authors:** Rick A van Dam, Alicia C Hogan, Andrew J Harford, Chris L Humphrey

**Affiliations:** ^1^ WQadvice Torrensville Australia; ^2^ Environmental Research Institute of the Supervising Scientist Australian Government Department of the Environment and Energy Darwin Northwest Territories Australia; ^3^ RMIT University Melbourne Victoria Australia; ^4^ Terrain Natural Resource Management Innisfail Queensland Australia

**Keywords:** Site‐specific, Water quality, Benchmark, Guideline value, Aquatic ecosystem

## Abstract

Existing prescriptive guidance on the derivation of local water quality benchmarks (WQBs; e.g., guideline values, criteria, standards) for protecting aquatic ecosystems is limited to only 3 to 4 specific approaches. These approaches do not represent the full suite available for deriving local WQBs for multiple types of water quality–related issues. The general lack of guidance is inconsistent with the need for, and benefits of, local WQBs, and can constrain the appropriate selection and subsequent evaluation of derivation approaches. Consequently, the defensibility of local WQBs may not be commensurate with the nature of the issues for which they are derived. Moreover, where local WQBs are incorporated into regulatory requirements, the lack of guidance presents a potential risk to the derivation of appropriate WQBs and the achievement of desired environmental outcomes. This review addresses the deficiency in guidance by 1) defining local WQBs and outlining initial considerations for deciding if one is required; 2) summarizing the existing regulatory context; 3) summarizing existing guidance and identifying gaps; 4) describing strengths, weaknesses, and potential applications of a range of derivation approaches based on laboratory and/or field data; and 5) presenting a conceptual framework for appropriately selecting and evaluating a derivation approach to best suit the need. The guidance incorporates an existing set of guiding principles for deriving local WQBs and reinforces an existing categorization of site‐adapted and site‐specific WQBs. The conceptual framework recognizes the need to strike an appropriate balance between effort and ecological risk and, thus, embeds the concept of fit‐for‐purpose by considering both the significance of the issue being assessed and the extent to which the approach provides confidence that the ecosystem will be appropriately protected. The guidance can be used by industry, regulators, and others for both the a priori selection and the post hoc evaluation of appropriate approaches for deriving local WQBs. *Integr Environ Assess Manag* 2019;15:683–702. © 2019 The Authors. *Integrated Environmental Assessment and Management* published by Wiley Periodicals, Inc. on behalf of Society of Environmental Toxicology & Chemistry (SETAC).

## Key Points

- Local water quality benchmarks (WQBs; i.e., guideline values, criteria, standards) are often used for regulatory purposes in numerous countries, yet the limited guidance for their derivation covers only 3 to 4 derivation methods.
- This review sets the global context for local WQB derivation, describes the strengths, weaknesses, and applications of a range of laboratory‐ and field‐based derivation approaches and, finally, presents a conceptual framework for selecting and evaluating a derivation approach to suit the need.
- Site‐adapted benchmarks are those in which a generic benchmark is modified to make it more representative of the site of interest, whereas site‐specific benchmarks are those in which a new benchmark is derived to specifically account for local conditions and communities.
- The conceptual framework embeds the concept of fit‐for‐purpose WQB derivation by considering 1) the significance of the issue being assessed and 2) the extent to which the derivation approach provides confidence that the aquatic ecosystem will be protected.

## INTRODUCTION

The provision of guidance is usually aimed at resolving a problem and, thus, helping those who are not fully familiar and experienced with a task to do it properly. Guidance helps to ensure competency and consistency in the application of a task across multiple parties and situations. Where guidance is lacking, clarity, quality, and even accuracy can be compromised, which can in turn potentially result in adverse outcomes. In the case of the derivation and use of local water quality benchmarks (WQBs, or their various global synonyms; see Table 1) for contaminants, poor guidance or a lack of guidance can potentially lead to inappropriate values that are underprotective and result in unintended environmental impacts, or that are overprotective and result in unnecessary management costs.

**Table 1:** Details of key terms used in this paper

Term as used in this paper	General definition	Synonyms (where applicable)
Benchmark	A numerical value for a contaminant that, if not exceeded, indicates a low risk that water quality and associated aquatic ecosystems will be unacceptably impacted.	Water quality guideline value (GV) Water quality criterion (WQC) Environmental quality standard (EQS)
Generic benchmark	A benchmark recommended for general application at any location in the absence of a more specific local benchmark.	Default GV Ambient WQC Generic EQS
Local benchmark	A benchmark that has been adapted or specifically developed for a site of interest (i.e., a site‐adapted or site‐specific benchmark).	
Site‐adapted benchmark	A generic benchmark that has been adapted, based on existing knowledge, to make it more relevant to a site of interest.	Modified default GV Site‐adapted GV
Site‐specific benchmark	A benchmark that has been specifically developed to account for relevant chemical, physical, and/or ecological conditions that occur at a site of interest.	Site‐specific GV Site‐specific WQC Site‐specific EQS

As we outline in the present paper, there is insufficient guidance on the selection and evaluation of approaches for deriving local WQBs for protecting aquatic ecosystems. Although we cannot pinpoint instances where this deficiency has resulted in an unacceptable management or environmental cost, evidence of the lack of guidance is clear among key end users, namely industry, who may be required to develop local WQBs, or regulators, who may be required to advise on and evaluate the appropriateness of local WQBs. We have encountered examples of poor understanding of the basis of local WQBs, including regulatory caution and inertia in even departing from generic WQBs, a lack of recognition of the difference between site‐adapted versus site‐specific WQBs (see Table 1), and a lack of recognition of the benefits of using best available science to derive local WQBs. These examples have contributed to the impetus behind our efforts to fill the gap in guidance for deriving local WQBs. We acknowledge that such guidance alone does not guarantee good quality WQBs and associated environmental outcomes but represents a key step in the path toward them.

van Dam et al. (2014) described and illustrated various approaches for deriving site‐specific WQBs, emphasizing 6 key guiding principles: 1) importance of understanding the issue; 2) ensuring the outcome addresses the issue; 3) need for robust derivation methods; 4) striking an appropriate balance between prescription and flexibility; 5) strengths of using multiple lines of evidence; and 6) importance of transparency and quality. The review and associated guidance provided here builds upon van Dam et al. (2014). We aim to help users make appropriate decisions about the a priori selection and post hoc evaluation of approaches for deriving local WQBs by highlighting their relative strengths and limitations and describing how they can be appropriately applied in water quality management, including regulation.

## CONTEXT

### Terminology

Although many of the concepts associated with WQBs are similar between countries, the terminology can differ considerably. For the purposes of clarity and consistency, Table 1 lists and defines the terms used in the present paper when applied in a general context. Although the terms “site‐specific WQB” and “local WQB” are often used interchangeably, here we have been more specific. Consistent with the Canadian Council of Ministers of the Environment (CCME 2003), we have defined 2 categories of local WQBs, site adapted and site specific, which are described in more detail in *Existing guidance and associated gaps* and *Strengths, limitations, and potential applications of water quality benchmark derivation approaches*. When citing specific examples, we use the specific terminology associated with that example (e.g., Canadian terminology for a Canadian example), although the need to ensure clarity and avoid confusion meant that doing so was not always possible. There are some key differences of usage and intent for WQBs among jurisdictions (e.g., some represent regulatory “pass/fail” numbers, whereas others represent guidelines only), and these are acknowledged and discussed where relevant to the guidance provided.

### What are local water quality benchmarks?

Water quality benchmarks are a tool for assessing potential impacts to aquatic ecosystems from contaminants and other stressors. For contaminants, they represent a measurable concentration below which there is considered to be a low risk of unacceptable effects occurring to the aquatic ecosystem (ANZG 2018a). In most cases, they should not be used alone but in conjunction with other lines of evidence (e.g., field biological monitoring), for assessing impacts of contaminants (USEPA 2016a; ANZG 2018a; Chapman 2018).

**Figure 1:**
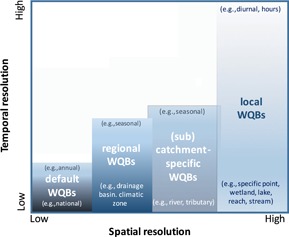
Different but overlapping spatial and temporal scales of water quality benchmarks (WQBs).

Although generic WQBs provide an important starting point for managing water quality, they cannot account for the large spatial and/or temporal variation in natural water quality, including variation in environmental variables that influence the bioavailability and, therefore, the toxicity of contaminants. Consequently, the past 2 decades have seen an increasing awareness of the need for local WQBs, with several jurisdictions (e.g., Australia and New Zealand, Canada) recommending them over generic WQBs wherever possible, and with some providing formal guidance on their derivation (see *Existing guidance and associated gaps*). In reality, there is an overlapping continuum of “types” of WQBs, based on both temporal and spatial factors, and more complex than the binary comparison of generic versus local WQB suggests. The different types of WQBs are depicted in Figure 1. Local WQBs typically are applied at a high‐resolution spatial scale (i.e., a specific location with specific biophysical characteristics) but can be applied across a large range of temporal scales, depending on the nature of the issue of concern. The United States Environmental Protection Agency (USEPA 1994a) considered that a site‐specific WQB (analogous to a local WQB in the context of the present paper) can apply across a spatial scale as large as a state, region, or watershed and/or catchment as long as the criteria for needing such a WQB are still met (see *Why and when we need local water quality benchmarks*). Although this is true, we would usually consider such values to be termed state, regional, or catchment WQBs, respectively, rather than site‐specific or local WQBs, noting there is some overlap (Figure 1).

Local WQBs aim to provide a significantly higher degree of confidence in the derived value than would generic WQBs, by taking into account local conditions that are considered relevant to the issue. Such local conditions could include environmental factors that are known to affect the behavior and/or bioavailability of the contaminants in question (e.g., hydrodynamic conditions that affect the pattern of occurrence of a contaminant; physicochemical conditions that affect contaminant speciation, partitioning, and/or interactions) and/or the local species or environments that are of particular relevance (e.g., being of specific ecological or economic value and/or thought to be particularly sensitive). The myriad possible combinations of biophysical characteristics at specific sites, and how these might influence contaminant toxicity, imply much complexity, which requires considerable knowledge and thought when determining 1) the need and 2) the most appropriate approach for deriving local WQBs.

### Why and when we need local water quality benchmarks

Local WQBs may be needed where generic WQBs are deemed likely not to provide an appropriate level of environmental protection, through either under‐ or overprotection. Typically, the need for a local WQB occurs where there is a contaminant of concern for which there is no generic WQB or the WQB is not appropriate and the protection of the aquatic ecosystem is a key management goal.

A generic WQB may not be appropriate or applicable to a site for a number of reasons, including these:

- The site is considered to be of high conservation and/or ecological value;
- there are local species and/or ecosystems of particular ecological, economic, and/or cultural importance present;
- the ambient water quality, including seasonal changes in water quality, has the potential to affect the bioavailability and the toxicity of the contaminant of concern;
- the nature of exposure of the receptors to the contaminant is markedly different from that typically used to derive generic WQBs (e.g., pulsed exposures); and/or
- natural background concentrations exceed the generic WQB.

In order to be able to make a decision about the necessity or otherwise of a local WQB, there must be a sound understanding of the nature of the contaminant issue, including the site characteristics. This need is elaborated upon in the following section.

### Decisions for deriving a local water quality benchmark

**Figure 2:**
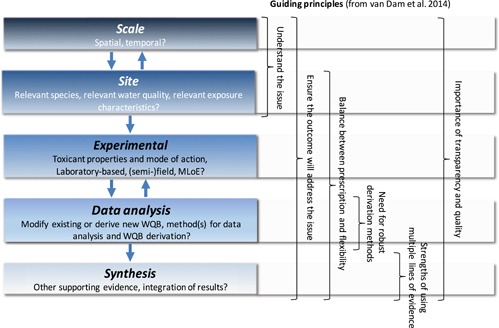
Aspects of, and associated decisions required for, deriving local WQBs. Depicted alongside these are the 6 guiding principles identified by van Dam et al. (2014). MLoE = multiple lines of evidence; WQB = water quality benchmark.

There are many considerations and subsequent decisions needed in order to derive an appropriate local WQB. These are summarized at a high level in Figure 2, along with their relationship to the key guiding principles identified by van Dam et al. (2014) and listed in the *Introduction*. In particular, the importance of understanding the issue, not only to determine whether a local WQB is needed but also to guide decisions on the most appropriate approach to deriving one, cannot be overemphasized. The most appropriate way to achieve this understanding is through stakeholder engagement and the subsequent development of conceptual models. Conceptual models are an important tool for aiding in the illustration and understanding of source–stressor–ecosystem receptor interactions and of specific conditions at a site, and substantial guidance on their value and development has been provided elsewhere (e.g., Suter 1996; Gross 2003; ANZG 2018a). The extent of their complexity should be commensurate with the scale of the issue being assessed and, thus, can range from a simple diagram or narrative to more complex system representations (Norton and Schofield 2017). For the purposes of deriving site‐specific WQBs, conceptual models need to capture the relevant spatial and temporal scales; the key ecological characteristics of the site, including water quality and potentially sensitive receptors (and how they might be affected); and the nature of the contaminants present at the site, including its exposure profile. Chapman and McPherson (2016) provide a good example of a conceptual model appropriate for assessing an effluent‐related contaminant issue.

The conceptual model should be used to guide decisions on the experimental approaches for collecting the data to derive the local WQB. In some cases, this work may include an impact assessment to help inform the derivation of the local WQB (see Argyle diamond mine example in *Weight‐of‐evidence process* and more detail in van Dam et al. 2014).

Highlighting the complexity of the decision‐making process, there are multiple approaches, ranging from simple to complex, that can be used to derive a local WQB (see *Strengths, limitations, and potential applications of water quality benchmark derivation approaches*). Very broadly, they can range from use of existing data to acquisition of new data; use of laboratory data to use of field data, or both; use of physical and chemical data and/or biological effects data; use of an accepted approach to use of a novel approach; use of a prescribed approach to use of a flexible approach; and combinations thereof. Given this complexity, it is pertinent to ask the question, how are regulators and industry meant to know what is, and is not, appropriate to the need or, in other words, what is fit‐for‐purpose?

### Regulatory context

It is of value to briefly consider the extent to which different jurisdictions encourage or require the derivation of local WQBs. The summary here is not intended to be exhaustive, but rather an example of the range of approaches adopted and a demonstration that local WQBs are considered a key component of water quality management and regulation in numerous jurisdictions. Acceptance of the use of local WQBs has imbued the regulatory framework in the United States for many years, where states can derive and apply local water quality criteria (WQC) if they deem ambient WQCs to be inappropriate (Carlson et al. 1984; Stephan et al. 1985; USEPA 1994a). In Australia and New Zealand, regulatory use of local guideline values (GVs) has been less formalized. However, the Australian and New Zealand Guidelines for Fresh and Marine Water Quality (ANZG 2018a) have recommended the use of local GVs over national default GVs since 2000, and as such, regulatory instruments such as waste discharge licenses often require that local GVs be derived and applied to assess compliance (e.g., Qld EHP 2009; Sinclair et al. 2014; Turner et al. 2016; Van Dam et al. 2018; NT EPA 2019). An exception to this is a national (generic) WQB (termed “attribute”) for nitrate in New Zealand fresh waters, which must not be exceeded (Ministry for the Environment 2018). In contrast to the United States, in Australia and New Zealand the onus on deriving local GVs falls mostly on the private sector rather than on the state. The situation in Canada is similar to that in Australia; local GVs are recommended over national generic GVs, and although they typically are not enforceable, they can be used to support the licensing and permitting of effluent discharges (CCME 2003). For example, site‐specific GVs are being applied in such a manner for a variety of mines across Canada and the Canadian oil sands (P Chapman, Chapema Environmental Strategies Ltd, North Vancouver, Canada, personal communication, 2016; e.g., Chapman and McPherson 2016). In South Africa, local WQC are recommended under certain conditions, and as with national criteria, they are not enforceable unless a legal document (i.e., act, regulation, or permit) explicitly requires that they be complied with (Department of Water Affairs and Forestry 1996; RSA DEA 2018). However, it was unclear from our review the extent to which this occurs.

The approach in Europe is somewhat different. Generic environmental quality standards (EQSs) are being developed for a list of specific priority substances, for application across all the European Union Member States. Compliance with the generic EQSs forms part of the assessment of ecological status for European waterways, while they are also used to set waste discharge permits to water bodies (European Commission 2011). To account for local water physicochemistry that might affect bioavailability (e.g., pH, DOC, hardness, alkalinity), the European Commission (2011) recommends using biotic ligand models (BLMs) where they exist (i.e., currently only for a number of metals, e.g., Cu, Ni, Zn). The bioavailability correction can be applied to either the EQS or the measured environmental concentrations.

It is very important that local WQBs are derived in a defensible manner and are fit‐for‐purpose because the process and associated decisions, which often are not straightforward, can affect the reputations of regulators and the regulated entities, as well as result in significant financial and environmental implications. The need for defensible, fit‐for‐purpose WQBs emphasizes the importance of having appropriate guidance for anyone who derives, uses, or assesses local WQBs (i.e., regulators, industry, and consultants who support both).

## EXISTING GUIDANCE AND ASSOCIATED GAPS

It is important to distinguish between methods for deriving generic WQBs and those for deriving local WQBs. Although there is much overlap between the two, there can (and should) be more flexibility when deriving local WQBs, especially site‐specific WQBs. Methods for generic WQB derivation typically require much prescription and standardization. Although there is often a need to use best professional judgment for certain decisions when deriving generic WQBs (see Warne et al. 2018), there are inherent risks associated with this (see Hahn et al. 2013). Although prescription is necessary to some extent for local WQBs, for example, in relation to data quality assessment, the fact that their intent is to account for local conditions of relevance, which will vary for different contaminants and sites, means that a single prescriptive method will rarely be applicable.

There is some stand‐alone guidance for deriving local WQBs, most notably that published by the USEPA (Carlson et al. 1984; USEPA 1994a) and Environment Canada (CCME 2003). These documents describe 3 and 4 specific procedures, respectively, for deriving local WQBs. The procedures are 1) a taxonomic composition adjustment termed the “recalculation procedure” (United States, Canada); 2) a bioavailability adjustment termed the “water‐effect ratio (WER) procedure” (United States, Canada); 3) the “resident species procedure,” effectively a combination of the recalculation and WER procedures (United States, Canada); and 4) a “background concentration procedure” (Canada only). Of these, the first two represent approaches for deriving site‐adapted WQBs, and the second two for deriving site‐specific WQBs.

The USEPA (1994a) provides guidance for states as to when local WQCs should be derived and, depending on the site's specific circumstances, which of the specified methods should be used. CCME (2003) includes guidance on the relative strengths and limitations of the 4 derivation methods, as well as examples of potential applications and a list of 14 rules for developing GVs using one or a combination of the methods. At least one of the methods, the WER procedure, has significant limitations and is typically no longer recommended for use by either jurisdiction (see *Strengths, limitations, and potential applications of water quality benchmark derivation approaches*). In both countries, procedures that differ from those formally recommended can be used to derive site‐specific WQBs if they can be demonstrated as being appropriate. However, no guidance is provided beyond that for the 3 to 4 specified procedures.

In Europe, the BLM is the only procedure recommended to be used to derive local EQSs (or, in fact, more often to predict bioavailable environmental contaminant concentrations for comparison with a generic Europe‐wide EQS [European Commission 2011]). Local field effects data typically are used to compare to, and validate the results of, the generic EQS (European Commission 2011).

In Australia and New Zealand, the recently revised Guidelines for Fresh and Marine Water Quality provide guidance for deriving GVs using either reference site data (similar to the background concentration procedure for Canada), laboratory effects data, (semi)field effects data, or a combination of these (ANZG 2018b), but only limited guidance on when local GVs might be required (ANZG 2018c; Warne et al. 2018). Some further guidance has been published specifically for the coal seam gas industry, on when and how to derive local GVs for toxicants and physicochemical variables (Huynh and Hobbs 2019). This guide follows the approaches recommended by ANZG (2018a) approach, with some additional guidance specifically tailored to coal seam gas water quality issues.

Guidance by other jurisdictions on deriving local WQBs is sparse or at least difficult to locate. The South African Water Quality Guidelines for Aquatic Ecosystems encourage the derivation of local GVs, but confine the acceptable approaches for doing so to the background (or reference site) concentration procedure and the WER procedure (Department of Water Affairs and Forestry 1996). Zhao et al. (2018) noted that the Chinese government recently initiated a revision of China's Environmental Quality Standards for Surface Water, and outlined a number of aspects in this process that are of priority, including greater emphasis on the development of site‐specific WQBs. China's current standards have no guidance on site‐specific WQBs, and it has been proposed that approaches similar to those used in the United States, in addition to methods such as the BLM, be adopted (Zhao et al. 2018). However, the extent to which such approaches will be adopted is unclear.

Published research proposing new refinements to methods for deriving WQBs is not uncommon (e.g., Fox 2010; Cormier and Suter 2013; van Dam et al. 2014; Peters et al. 2014; Wang et al. 2015). Promisingly, some approaches have found their way into formal guidance documents. For example, the USEPA (2016b) published a field‐based criterion for electrical conductivity, which also provides guidance on the derivation approach. Also, emerging approaches for deriving site‐specific WQBs (mostly based on field effects data), summarized by van Dam et al. (2014) and Chariton et al. (2016), have been highlighted in ANZG (2018d). Nevertheless, much of the formal guidance remains either limited to only a handful of methods or is general in its descriptions, and there is currently no comprehensive synthesis of the multiple methods available for deriving local WQBs. Although van Dam et al. (2014) described examples of physicochemical, ecotoxicological, and ecological approaches for deriving site‐specific GVs that were consistent with the Australian and New Zealand guidance, there is still very little guidance on the appropriate selection, and/or evaluation of the rigor, of the various methods. The need for such guidance is especially important given the increasing acceptance and use of additional approaches such as those mentioned in this review.

## STRENGTHS, LIMITATIONS, AND POTENTIAL APPLICATIONS OF WATER QUALITY BENCHMARK DERIVATION APPROACHES

The multiple approaches for deriving local WQBs fall into 2 categories: 1) where an existing, usually generic, WQB is modified—termed a “site‐adapted WQB”; and 2) where a new WQB is derived on the basis of site‐specific data—termed a “site‐specific WQB” (see Table 1). Table 2 summarizes the strengths and limitations of the most common derivation approaches for the 2 categories. It builds on similar tabulated advice from CCME (2003) for the 4 types of local WQB derivation procedures recommended in Canada. Although not exhaustive, the approaches listed in Table 2 generally encompass the majority of approaches, or variations thereof, that can be considered and used for local WQB derivation. In describing each of the approaches, an assumption was made that important aspects such as experimental design, use of ecologically relevant measurement endpoints (e.g., growth, development, reproduction, survival), sampling, storage, transport and analysis of contaminants, statistical treatment of the toxicity data, and where relevant, minimum sample sizes for the use of species sensitivity distributions (SSDs) meet the requirements for the derivation of rigorous WQBs; hence, they are not the focus of the present review.

**Table 2:** Key attributes of approaches that can be used to derive site‐adapted or site‐specific water quality benchmarks

Approach	Scientific defensibility		Applicability				Practicality			Cost effectiveness	
	Based on biological effects data?	Considers site‐specific conditions?	Applicable to all classes of chemicals?	Degree of site‐specificity	Requires specific characterization of toxicity modifying factors?	Uncertainty in the applicability	Supports the development of numerical benchmarks?	Level of complexity	Timeliness	Requires generation of new biological data?	Relative cost of development
Modification of existing generic benchmark (site‐adapted)											
Added risk approach	No	No	No^a^	Low	No	High	Yes	Low	Fast	No	Low
Recalculation procedure (species removal)	Yes	No^b^	Yes	Low	No	High	Yes	Low	Fast	No	Low
WER approach	Yes	Yes	Yes	Moderate	No^c^	Moderate	Yes	Moderate	Moderate	Yes	Moderate
Adjustment based on 1–2 modifiers of toxicity	Yes	Yes	No^d^	Low to Moderate	Yes	Moderate	Yes	Low to Moderate	Fast	No^e^	Low^e^
Adjustment based on >2 modifiers of toxicity (BLM, MLR)	Yes	Yes	No^f^	High	Yes	Low to Moderate	Yes	Moderate to High	Fast^g^	No^g^	Low^g^
Derivation of new site‐specific benchmark (site‐specific)											
Background or reference condition approach	No	Yes	No^h^	High	No	Low	Yes	Low	Fast^i^	No	Low
Nonlocal species in local water quality	Yes	Yes	Yes	Moderate	No^c^	Moderate	Yes	Moderate	Moderate	Yes	Moderate
All local species in local water quality	Yes	Yes	Yes	High	No^c^	Low	Yes	Moderate	Moderate	Yes	Moderate to high
All local species in local water quality supplemented with other relevant data	Yes	Yes	Yes	High	No^c^	Low	Yes	Moderate	Moderate	Yes	Moderate to high
All local species in local water quality with adjustment based on data for local toxicity modifying factors	Yes	Yes	Yes	High	Yes	Low	Yes	Moderate to High	Moderate to Slow	Yes	High
Use of field or semifield data	Yes	Yes	Yes	High	No^c^	Low^j^	Yes	High	Moderate to Slow	Yes	High
WoE approach	Yes	Yes	Yes	High	Possibly	Very low	Yes	High	Slow	Yes	Very high

BLM = Biotic ligand model; MLR = Multiple linear regression; WER = water‐effect ratio; WoE = weight‐of‐evidence.

^a^ The approach is relevant only to naturally occurring chemicals.

^b^ The approach does not consider site‐specific conditions other than the local relevance of the species.

^c^ The approach characterizes the combined effect of, rather than individual, toxicity modifying factors present in the local waters at the time of sampling.

^d^ The approach is currently limited to chemicals for which the relationship between modifying factor and toxicity has been quantified (i.e., some metals and nonmetallic inorganics).

^e^ Assuming the relationships quantifying the effects of the modifying factor on the chemical in question have already been developed.

^f^ The approach is currently limited to some metals.

^g^ Assuming the BLM or MLR for the chemical in question is already developed and available as an online tool.

^h^ The approach is applicable only to naturally occurring chemicals and possibly to some globally distributed anthropogenic chemicals.

^i^ Assuming an appropriate monitoring data set already exists.

^j^ Assuming the criteria for being suitable for deriving site‐specific benchmarks have been met.

### Approaches for deriving site‐adapted water quality benchmarks

#### Added risk approach

The added risk approach (ARA) was first implemented in the Netherlands in the 1990s to incorporate the background concentration of naturally occurring substances, particularly metals, in environmental risk limits (Crommentuijn et al. 1997). It involves a maximum permissible addition (MPA) of the substance that is applied to the background concentration, to derive a maximum permissible concentration (MPC), which is, effectively, a site‐adapted WQB. The MPA is typically the maximum amount of substance that may be added to the background concentration of the substance without adversely affecting the assessed ecosystem, and is usually equivalent to a generic WQB that has been based only on the concentrations of substance added to the toxicity tests, by subtracting the test medium background concentrations from the total measured test concentrations prior to the analyses (European Commission 2011). The ARA prevents the WQB from being less than the natural background concentration, for example, in waters where the substance is present at naturally elevated concentrations. However, as noted by the European Commission (2011) and the ANZG (2018a), the relationship between toxicity and natural background concentrations is unknown, and some populations might in fact live close to their upper tolerance limit, particularly in highly mineralized areas, where metal concentrations are naturally high and the ecosystems can already be naturally stressed to some degree.

The ARA has gained little traction in Australia, New Zealand, Canada, and the United States. In Australia, New Zealand, and Canada, the preferred approach when natural background concentrations (of naturally occurring substances) exceed generic WQBs is to derive site‐specific WQBs based on a specific percentile of the natural background or reference condition data (see *Reference condition approach*; CCME 2003; ANZG 2018e). In contrast, the ARA is used in Europe, as an option to derive site‐adapted EQSs for metals where natural background concentrations exceed the generic EQS (European Commission 2011). If using the ARA, it is imperative that the spatial and temporal variability in background concentrations is well characterized. With the increasing development of BLMs and multiple linear regression models to derive bioavailability‐based WQBs for metals (see *Adjustment based on >2 modifiers of toxicity*), it is unlikely that the ARA will be more widely employed. Even in cases where information on bioavailability and toxicity modifying factors is limited, site‐specific approaches are preferred.

#### Recalculation procedure

The recalculation procedure involves the removal of data for species not local or relevant (e.g., as a surrogate of local species) to the site of interest from the data set that was used to derive the generic GV for the contaminant of interest. According to CCME (2003), local species are those that are or in the past were, usually, seasonally or intermittently present at the site. A site‐adapted GV is then derived from the modified data set that purports to better reflect the taxonomy and, therefore, the sensitivity of species that reside at the site of interest. The approach is recommended for use in both the United States (Carlson et al. 1984; USEPA 2013a) and Canada (CCME 2003) but can potentially be applied elsewhere. A formal and transparent decision process is needed to ensure that species are appropriately retained in, or removed from, the data set, as detailed by the USEPA (2013a) and the CCME (2003).

Assuming appropriate rules are in place, the recalculation procedure is generally simple, defensible, and intuitive, although it does have several limitations (Carlson et al. 1984; CCME 2003; USEPA 2013a). Additional field surveys may be needed to comprehensively identify resident species at the site of interest. Also, given that original data sets for generic WQBs are often small, removal of even a small quantity of data can reduce the data set size to below that required by the relevant jurisdiction to use an SSD approach for WQB derivation. This problem is especially relevant in countries outside Europe and North America, where many of the standard toxicity test species are not local (although may be deemed relevant). Where the adjusted data set is small, there may be a need for additional toxicological information on resident species to ensure the adequacy of the data set used to derive the site‐adapted WQB.

#### Water‐effect ratio approach

The WER approach is a method for modifying generic WQBs by accounting for the receiving water characteristics that might influence the toxicity of the contaminant of concern. Little knowledge of the factors affecting toxicity, or their mechanisms, is required to use the approach. The WER approach has been formalized for local WQB derivation in both the United States and Canada (USEPA 1994b; CCME 2003).

The WER approach involves conducting parallel toxicity tests using 2 different test diluents, a standard laboratory water and water collected from the site of interest (USEPA 1994b; CCME 2003). The difference in toxic response observed between the 2 diluents provides a measure of the ability of the site water to modify the toxicity of the contaminant of concern. The WER is calculated by dividing the toxicity estimate for a particular percent effect (e.g., the LC50, EC50, or inhibitory concentration [IC50]), observed in the site water test, by the estimate generated from the standard laboratory water test. The generic WQB is then modified by multiplying it by the WER (USEPA 1994b; CCME 2003).

Notwithstanding the simple concept of the WER approach, several factors need to be considered for its implementation. These include accounting for the spatial and temporal variability of the site water chemistry, frequency of reevaluation of the calculated WERs, choice of a standard laboratory water composition, and test species selection and acclimation. Details of, and guidance on, these issues are provided by the USEPA (1994b) and Welsh et al. (2000). It is important to note that these issues also apply to other approaches that involve use of local waters and species for deriving local WQBs.

There appears to be some recent movement away from the routine use of the WER approach, particularly for toxicants such as cationic metals, where the factors affecting toxicity are well understood. The USEPA (2007) stated that the use of the BLM (see *Adjustment based on >2 modifiers of toxicity*) for Cu was preferable over the “costly and time consuming” WER approach. This is a reasonable position where there is confidence in the accuracy of the BLM or other predictive models for a particular contaminant and site. Nevertheless, the WER approach may still be of relevance for some situations in which knowledge about toxicity modifying factors is limited and a site‐specific WQB based on local species data is not necessary. Bao et al. (2018) provide a recent example of the use of the WER approach, for Cu in marine ecosystems of Hong Kong.

#### Adjustment based on 1 or 2 modifiers of toxicity

For some contaminants, adjustments can be applied to generic WQBs based on quantified relationships between toxicity and 1 or 2 key toxicity modifying factors. However, these adjustments are available for only a very limited number of, primarily inorganic, contaminants.

Adjustments for water hardness are available for a number of metals, namely Cd, Cr(III), Cu, Pb, Ni, and Zn (Warne et al. 2018). However, their appropriateness has been questioned due to 1) the limited, fish‐dominated, acute toxicity data upon which the relationships were established and 2) the fact that hardness is just one of several important toxicity modifying factors for these metals (e.g., Markich et al. 2005; Merrington et al. 2016). Not surprisingly, the USEPA (2007) stated that the use of a multiple parameter bioavailability model such as the BLM (see next section) for Cu would result in a more appropriate WQC than would the hardness adjustment approach. As a result of the abovementioned limitations, the ANZG (2018a) removed Cu from the list of metals for which hardness corrections could be applied.

Ammonia is another common contaminant for which adjustments can be made to a generic WQB. Algorithms have been developed to adjust the generic WQB according to the pH and temperature of the surface waters at the site of interest (USEPA 2013b). This approach for ammonia has received less criticism than have the hardness corrections for metals, probably due to the fact that pH and temperature are the predominant toxicity modifying factors for ammonia. However, application of the ammonia adjustments still has its limitations, as recently noted by Mooney et al. (2018, 2019). These limitations relate to 1) the limited basis of the algorithms, having been developed for just a small suite of (fish‐dominated) organisms, and 2) the potential influence of other toxicity modifying factors, such as ionic strength.

The ability to adjust a generic WQB for just 1 or 2 toxicity modifying factors is a quick and simple way to improve the relevance of the WQB to the site of interest. However, confidence in the adjusted WQB may still be relatively low. The following section discusses methods for taking into account more than 2 toxicity modifying factors.

#### Adjustment based on >2 modifiers of toxicity

This section considers the BLM and, to a lesser extent, multiple linear regression models (MLRs), both of which have been applied to only a limited number of metals.

The BLM is a mechanistic model that relates receiving water physicochemistry to the bioavailability and toxicity of metals to aquatic organisms (Di Toro et al. 2001; Paquin et al. 2002). The BLM concept is based on the principle that toxicity occurs once the amount of metal bound to “biotic ligands” on the water–organism interface exceeds a certain threshold (Di Toro et al. 2001). The BLM framework consists of a geochemical equilibrium model that accounts for speciation and competitive effects on metal ions in the receiving environment, and a gill surface interaction model (or similar) that estimates the amount of metal bound to the site of toxic action on or in the organism (Di Toro et al. 2001; Paquin et al. 2002).

The gill has been shown to be the primary binding site for metals in fish, and, as such, much of the early developmental work on the BLM was undertaken using 2 fish species (rainbow trout and fathead minnows) (Paquin et al. 2002). However, because fish generally are less sensitive to metals than other taxa (e.g., cladocerans and unicellular algae), and these more sensitive taxa are used in generic WQB derivations, the need to extend the BLM to other organisms was recognized early in the development of the BLM (Niyogi and Wood 2004).

It was hypothesized that biotic ligands apply to all aquatic organisms (Di Toro et al. 2001), and several studies discussed by Niyogi and Wood (2004) showed that 1) measurement of toxicity was an acceptable proxy for determining a binding affinity based on gill metal binding, and 2) surface‐bound metals and/or whole body metal burdens correlated well with toxic responses. These relationships enabled gill–metal affinity constants to be derived directly from toxicity data for the recalibration of the fish BLM to organisms that are too small for direct measurement of gill metal burdens or do not possess gills.

Because the aim of most WQBs is to protect organisms from long‐term exposures that can occur throughout their life cycle, predictions of chronic toxicity, rather than acute toxicity, are considered most relevant for generic WQB adjustment (Niyogi and Wood 2003). The need for BLMs that predict the toxicity of chronic metal exposures has long been recognized, and chronic BLMs have been developed for Cu, Pb, Mn, Ni, and Zn (Heijerick et al. 2002; De Schamphelaere and Janssen 2004; Peters, Lofts et al. 2011; Peters, Merrington et al. 2011; Nys et al. 2014). Although the focus has been primarily on freshwater ecosystems, BLMs have also been developed for the marine environment (e.g., USEPA 2016c).

The primary benefits of the BLM approach over other means of adjusting generic WQBs is the large number of toxicity modifying factors that are considered in the model (e.g., pH, DOC, hardness (i.e., Mg and Ca), alkalinity, temperature and major ions (i.e., Na, sulfate, K, and chloride in the Cu BLM [USEPA 2007]). Although these factors are inherently incorporated into empirical site‐specific toxicity testing approaches (see *Approaches for deriving site‐specific benchmarks*), they are dynamic in the environment, and the BLM allows for predictions of toxic effects to be updated each time new physicochemical information is collected. Thus, use of the BLM is more time and cost effective than rerunning site‐specific toxicity tests (and herein lies its appeal). Considerable research has been undertaken in Europe, Canada, and the United States to develop user‐friendly tools for routine use in environmental regulation (e.g., HydroQual 2007; Bio‐met 2017).

Various assumptions are made that can limit the accuracy of the BLM predictions. Many of the BLM assumptions relate to changes in membrane physiology and uptake kinetics during exposure (see Campbell et al. 2002; Paquin et al. 2002; Hassler et al. 2004; Niyogi and Wood 2004; Slaveykova and Wilkinson 2005). Another limitation raised by Nys et al. (2014) is the lack of understanding of the nature of different forms of DOC and a subsequent inability to adjust the geochemical speciation component of the BLM to account for dissolved organic matter that is more or less able to ameliorate toxicity. Further, BLMs are still based on the responses of a small number of species from 3 taxonomic groups (i.e., fish, crustaceans, and algae). This falls short of the minimum number of species or taxa required by most jurisdictions for WQB derivation.

In recognition of the abovementioned limitations, BLMs are validated using synthetic and natural waters prior to being considered acceptable for broader use, with their acceptability being assessed by determining the proportion of predictions (based on test water chemistry) that fall within a factor of 2 of the actual observed toxicity (McLaughlin 2015). The literature suggests that a BLM is considered acceptable if more than 90% of the predictions fall within a factor of 2 of the observed toxicity (Bury et al. 2002; Niyogi and Wood 2004; Peters, Lofts et al. 2011; Nys et al. 2014). Generally, BLM validation studies have been undertaken for single metal exposure scenarios; therefore, the accuracy of BLM predictions for metals discharged within complex mixtures is not well understood. Results from studies examining the accuracy of BLM predictions of metal mixture toxicity indicate that the BLM is useful for predicting the toxicity of binary metal mixtures (Jho et al. 2011) and mixtures of metals that share a common mode of action (Kamo and Nagai 2008; Iwasaki et al. 2015).

Another approach to modeling the influence of toxicity modifying factors on WQBs for metals is through the use of MLR equations (Brix et al. 2017). Correlational relationships between toxicity and multiple key toxicity modifying factors are developed for single species, with the relationships then being used to adjust a WQB depending on the water quality characteristics. The approach has recently been adopted by Environment Canada for Zn in fresh waters (CCME 2018) and proposed by the USEPA for Al in fresh waters (USEPA 2017; based on the work by De Forest et al. 2018). The MLR approach is similar but less complex than the BLM approach. For this reason, it may become a preferred approach for adjusting generic WQBs over the BLM.

Overall the BLM and MLR approaches have been demonstrated to be useful tools for modifying generic WQBs for a limited suite of single metals and, for the BLM, also simple metal mixtures in fresh waters. Further research will extend these modeling approaches to a wider range of metals in both freshwater and marine environments, although it may prove difficult to apply the BLM theory to metals with multiple routes of exposure, multiple mechanisms of action, and trophic level effects. Effective and appropriate use of such models for environmental management will be dependent on practitioners understanding their limitations and the physico‐chemical and biological conditions under which they have been validated, so that the approach is applied only to scenarios that fall within the bounds of the models.

### Approaches for deriving site‐specific water quality benchmarks

In some cases, modification of a generic WQB will not be possible or sufficient for the situation, and it might be necessary to fully derive a new, site‐specific WQB. A site‐specific WQB can be based on existing reference or background water quality data or on data generated by laboratory and/or field‐based effects assessments.

#### Reference condition approach

Site‐specific WQBs derived from water chemistry data collected before significant human disturbance and/or from reference sites quantify the background range of naturally occurring toxicants in a waterway. Their derivation generally involves using data from baseline or minimally disturbed sites to generate percentiles of data that best reflect the reference data distribution. They are recommended in various jurisdictions (e.g., see Carlson et al. 1984; CCME 2003; ANZG 2018a). In Australia and New Zealand, the 80th centile (or, in the case of physicochemical variables such as temperature and pH, also the 20th centile) of the reference data is recommended as a site‐specific GV for “slightly‐to‐moderately disturbed” systems which, for naturally occurring compounds, is generally preferred over a default GV (ANZG 2018e). Its application involves comparing the median (50th centile) of the test site data to the reference site 80th centile. The recommendation of this specific comparison was to simply quantify the notion of a “measurable perturbation” at the test site. Although the percentiles at the basis of this comparison were largely arbitrary selections, the approach has retained its value and appeal among experts. However, the comparison can be adjusted, depending on the management goals or nature of the receiving waters. For example, for more highly valued ecosystems or surface waters low in ionic strength or seasonally (and naturally) stressed, the comparison could be more conservative, such as comparing the reference site 60th or 70th centile to the test site median, or even not allowing any change in water quality (i.e., reference site median vs test site median).

Although this approach identifies a change from the natural reference condition, it does not provide information on the biological or ecological ramifications of an exceedance, or even a nonexceedance, of the site‐specific WQB (van Dam et al. 2014). Thus, in Australia and New Zealand, the ANZG (2018e) recommends the use of local reference chemistry data for deriving site‐specific GVs only in the absence of local biological effects data. van Dam et al. (2014) describe the derivation and application of a reference‐based site‐specific GV for Mn in a freshwater stream.

Site‐specific WQBs based on reference water quality data are most useful when natural concentrations of a substance are above the generic WQB because they recognize that the aquatic ecosystem has evolved in the presence of naturally elevated concentrations of the contaminant. The assumptions are that the physicochemistry of the site water reduces the bioavailability and, hence, the toxicity of the contaminant, or the resident biota have adapted to the presence of the contaminant over time. Where receiving site concentrations exceed both generic WQBs and site‐specific WQBs based on reference water quality, it is considered prudent to further investigate the potential for ecological impacts, through site‐specific toxicity testing and/or field monitoring/assessment of biological communities.

#### Nonlocal species in local water quality

Most toxicity tests are conducted following standardized methods that optimize the health and performance of test organisms and generate reproducible results. Several years of research and development are usually required to produce such methods. Consequently, it is not always practicable or necessary to invest the resources to develop methods for local species. As such, a limited suite of freshwater test organisms is currently used in commercial ecotoxicology testing, and a selection of these species may be used for site‐specific WQB derivations based on their “regional (including climatic) relevance,” rather than using local organisms for which protocols have been developed. These species may or may not occur at the site of interest, and are effectively used as surrogates of local species. Examples of the use of locally relevant, but not locally collected, species to derive site‐specific WQBs are described in Chapman and McPherson (2016) and Chen et al. (2016).

Local (uncontaminated) site waters are used as the diluent, typically instead of a standard synthetic or reconstituted water, to account for the effects of toxicity modifying factors associated with the local water quality. It is important that the physicochemical characteristics of the site water fall within the tolerance ranges of the test organisms. Related, a pretest acclimation period (e.g., of 2 wk) in the site water provides test organisms the opportunity to physiologically adjust to the water quality, which should promote better test organism performance during testing (Soucek and Kennedy 2005; van Dam et al. 2014). As with all site‐specific WQBs based on laboratory toxicity testing data (i.e., the current approach and for all such approaches described below), minimum data requirements prescribed by the relevant jurisdiction should be met. These are typically set to enable the use of an SSD approach for deriving the WQB.

For some assessments, a hybrid approach between the use of nonlocal and local species (see *Local species in local water quality*) may be desirable, for several reasons: 1) replacing some standard species that cannot tolerate the local water quality with similar local species; 2) selecting several (e.g., 2–3) local species taxonomically similar to a selection of the standard species to validate the use of a larger suite of standard species as surrogates of the local species; or 3) where there is a particularly important local species. By using local species that have an “analogous” standard species (e.g., a congener), the test method for the standard species can potentially be used, sometimes with minor modifications, greatly minimizing the extent of additional method development (e.g., van Dam et al. 2014). A good example of the inclusion of a local species due to its (ecological) importance is the development and application of a toxicity test method for the giant cuttlefish, *Sepia abama*, for deriving a site‐specific WQB for brine discharges from a proposed desalination plant (Dupavillon and Gillanders 2009). In this case, the proposed brine discharge was to be in the vicinity of an internationally significant breeding ground for this species. The resultant toxicity data were combined with data for nonlocal, but regionally relevant, species to derive a site‐specific WQB (Warne et al. 2010).

#### Local species in local water quality

Where the water quality issue is of sufficient significance, site‐specific WQBs should take into account not just the specific water quality characteristics of the site, but also the sensitivity of local species. This approach will require the development of new, or the adaptation of existing, test methods for local species. Such WQBs will provide greater confidence of adequate environmental protection. As noted for the previous derivation approach, the development and maintenance of toxicity test methods for local species requires significant investment. Such investment may not be justifiable for “one‐off” site‐specific assessments or where the contaminant issue is not of sufficient significance (see *Guidance for selecting and assessing approaches*).

Typically, enough local species across a range of taxonomic groups should be tested so as to enable the use of an SSD approach for deriving the site‐specific WQB. Where fewer local species are tested, for example where 1 or 2 species are of particular economic, ecological and/or cultural importance, this would typically represent a validation exercise for the generic WQB, to ensure it is sufficiently protective of the species tested. Any subsequent revised WQB based on inclusion of the newly‐generated toxicity data for the 1‐2 species in the overall generic WQB toxicity dataset would be considered a site‐adapted WQB rather than a site‐specific WQB, because it is still predominantly based on generic toxicity data rather than site‐specific toxicity data.

A notable example of the need to develop site‐specific toxicity test methods is that of Ranger uranium (U) mine in northern Australia (see van Dam et al. 2002). A 1978 federal act (Commonwealth of Australia 1978) established an agency that would ensure the highly valued ecosystems of the Alligator Rivers Region were protected from the potential impacts of the Ranger mine and other U mining activities in the region. In parallel, the 20 000 km^2^ of largely undisturbed landscape surrounding the various mining leases was declared Kakadu National Park, which was later afforded World Heritage and Ramsar listings. The act included the establishment of a research institute to study the region's ecology and also to develop standards and procedures to ensure environmental protection. As part of this institute, an ecotoxicology facility was established to develop and use toxicity test methods for local species in local receiving waters in order to assess mine water toxicity and to develop water quality limits for contaminants of potential concern. Over the past 30 years, well over 20 species have been assessed for their suitability as toxicity test species, with full methods having been developed for at least 8 species. The large investment in site‐specific ecotoxicological methods, as well as in other relevant scientific disciplines, has been in recognition of the highly valued ecosystems surrounding the mine, as well as the subsequent identification of contaminants with significant risk profiles. Some examples of the use of toxicity data for local species in local water to derive site‐specific WQBs for single contaminants for the Ranger mine are described in Harford et al. (2015), Mooney et al. (2019), and van Dam et al. (2010, 2017).

In an attempt to include more local species in site‐specific assessments without the need for time‐consuming and expensive test development, a rapid toxicity assessment approach has been developed whereby short‐term (acute) tests are undertaken on many organisms directly collected from the environment of interest (Kefford et al. 2005). The approach places less emphasis on the need to adhere to typical experimental requirements, such as standardized methods, minimum sample sizes and replication, organism acclimation, and chronic exposures, on the basis that sensitivity can be quickly and approximately estimated for many species. Although this approach has the benefit of generating data for a larger set of species that is a better representation of the actual aquatic community of interest, it is also limited in that only acute toxicity is assessed, data variability can be high (due to less standardized test conditions, variable organism responses, and small sample sizes), and it typically requires the use of a safety factor to arrive at a (chronic) WQB. Consequently, careful thought should be given to the appropriateness of this approach for the contaminant issue in question. A recent example of an appropriate use of the rapid testing approach is provided by Kefford et al. (2019) for Antarctic marine invertebrates that are extremely difficult to culture and undertake chronic toxicity testing with in the laboratory.

#### Local species in local water quality supplemented with other relevant data

Site‐specific WQBs derived from local species data often are based on small sample sizes (e.g., <7), resulting in statistical uncertainty and undermining confidence in the WQB. It is possible to supplement site‐specific data sets with data for nonlocal species in nonlocal water quality where the data have sufficient relevance. Such data would typically have been generated under environmental conditions (i.e., water physicochemistry) similar to those at the site of interest, and where the bioavailability of the contaminant would be considered to be similar to that in the environment of interest. The inclusion of nonlocal data should be clearly justified, and it would be prudent to document and “reality check” the effect of the additional data on the site‐specific WQB. Harford et al. (2015) derived a site‐specific WQB for Mn from an SSD based on toxicity data for 6 local species in local receiving water and 3 nonlocal species that had been tested under similar water quality conditions. The inclusion of the nonlocal data resulted in an SSD with greater certainty in its estimates and, therefore, a more reliable WQB without compromising its site specificity.

#### Local species in local water quality with adjustment based on data for local toxicity modifying factors

Physicochemistry of water bodies that influences contaminant bioavailability is often spatially and temporally variable, potentially making it difficult to fully characterize site‐specific toxicity and derive appropriate WQBs. In certain situations, it may be necessary to quantify the influence of key toxicity modifying factors on toxicity of a contaminant to local species (rather than relying on such data for nonlocal species and conditions) and to incorporate these relationships into the site‐specific WQB. This approach greatly increases the amount of research required but offers significantly increased confidence in the resulting site‐specific WQB. Therefore, the cost–benefit associated with this approach needs to be considered by decision makers. For example, a site‐specific WQB for U derived by van Dam et al. (2017) incorporated the influence of DOC on U toxicity because it was known to be a key toxicity modifying factor. Although water hardness and alkalinity are also important modifiers of U toxicity, they were not relevant in this case due to their consistently low levels in the site waters. For the same site, a site‐specific WQB for Mg was developed that first incorporated the influence of Ca concentration (van Dam et al. 2010) and, subsequently, exposure duration, on Mg toxicity (Hogan et al. 2013).

#### Use of field or semifield data

Although laboratory toxicity testing is the most commonly used approach for generating data to derive site‐specific WQBs, it is limited in both the number of species that can be practically assessed and the range of (important) natural and variable environmental conditions that can be simulated. Such standard testing is also less useful for stressors that are indirect (e.g., nutrients), that are without a toxic chemical mode of action (e.g., deposited sediment), or that bioaccumulate over extended exposure periods (e.g., persistent organic pollutants). Further, some important and sensitive species cannot be readily cultured and/or tested under laboratory conditions (e.g., many aquatic insects), while such testing cannot account for ecological interactions (see Cormier et al. 2008; European Commission 2011). The use of field or semifield studies offers opportunities to overcome such limitations.

Field exposures in natural systems provide the greatest environmental realism for deriving site‐specific WQBs. However, they are infrequently used to derive site‐specific WQBs. What is gained from a field study in terms of environmental relevance is often lost in terms of cause and effect (with the opposite applying to laboratory exposures). Relevant international agencies are mixed in their positions on field effects studies. The European Commission (2011) and the CCME (2007) noted that field observational studies may be too confounded to be useful in WQB derivations, although the derived data are viewed as important in evaluating and validating the final WQB. In contrast, in Australia and New Zealand (ANZG (2018d), field effects data are considered complementary or potentially even preferred over laboratory toxicity testing data for WQB derivation. Also, in the United States, Cormier and Suter (2013) and the USEPA (2016b) have published methods for deriving field‐based WQBs for specific conductivity. This protocol has already been applied and tested for conductivity in Appalachian mountain streams related to coal mining activities (Cormier, Suter, and Zheng 2013), as well as several other regions (USEPA 2016b). These recent efforts in the United States have focused on the development of methods to disentangle effects of confounding stressors. The outcome is greatly enhanced inference and evidence of cause and effect of stressors, which are difficult to study under laboratory conditions (Cormier, Suter, Zheng, and Pond 2013; Suter and Cormier 2013; Coffey et al. 2014).

For field effects studies to be employed for deriving site‐specific WQBs, acceptable quality and strength of evidence must first be demonstrated. In terms of the strength of evidence, the appropriateness of a field effects study for deriving a site‐specific WQB can largely be determined by the following 3 factors: 1) the degree of confounding from stressors or environmental variables other than the contaminant of interest, 2) the adequacy of the contaminant exposure gradient, and 3) the number, and magnitude of response, of biological assemblages assessed, especially those known to be sensitive to the contaminants of concern. Thus, the most suitable field effects data for deriving a site‐specific WQB would be those from a study in which the confounding was low (or can be resolved), the exposure gradient was sufficient to elicit a range of biological responses, and communities of potentially sensitive organisms were assessed. Warne et al. (2018) provided an expanded list of criteria for accepting field effects data that essentially encompasses the above 3 factors, and which represents a combination of the criteria listed by the Organisation for Economic Co‐operation and Development (OECD 1992) and the European Commission (2011). Perceval et al. (2011) and Buchwalter et al. (2017) have also recently promoted and discussed the merits of using field data for informing or deriving WQBs.

Mesocosm studies can represent a middle ground between field and laboratory exposures by combining beneficial elements of both approaches (Buchwalter et al. 2017). Although mesocosm and microcosm studies are often discussed together, the former are generally larger studies in field or semifield environments with relatively complex biological communities, whereas the latter are typically much smaller, laboratory‐based studies with significantly less biological complexity. For this reason, microcosm studies are unlikely to be a sole source of data for WQB derivation, but could be used with other laboratory‐ and/or field‐based lines of evidence. If designed well, mesocosm studies can be used for deriving site‐specific WQBs, because they offer:

- control for various environmental variables and the capacity for experimental replication not often possible in field studies in natural settings;
- the ability to establish concentration–response relationships for complex, community‐ and/or ecosystem‐level responses;
- measures of both direct and indirect responses across multiple taxonomic communities (e.g., phytoplankton, diatoms, zooplankton, macroinvertebrates); and
- greater representativeness of reality than laboratory tests because they can be performed at larger spatial and temporal scales under locally relevant conditions.

However, to be applicable for use in deriving WQBs, mesocosm studies would need to meet the same quality and strength of evidence criteria for field studies listed earlier in this section. Moreover, the mesocosm system should be designed to reflect the basic characteristics of the ecosystem of concern (Shaw and Kennedy 1996). Certainly, mesocosm studies have their own limitations, including often high variability, expense, and being logistically complex, which often results in only a few exposure concentrations being tested. There are few published examples of the use of mesocosm data to derive WQBs, which perhaps suggests that the limitations outweigh the strengths—or there is that perception at least. It seems that the more common and appropriate use of mesocosm study data is in a weight‐of‐evidence (WoE) approach, to support other data in WQB derivation and/or, more broadly, in risk assessments (Shaw and Kennedy 1996; European Commission 2011; Buchwalter et al. 2017). Clements and Kotalik (2016) undertook a series of mesocosm experiments to assess the USEPA aquatic life criterion for conductivity. Although not a WQB derivation exercise, the data could have been used to derive WQBs for the salts tested.

Analytical methods from other fields in ecology and biology disciplines are being increasingly recognized as being applicable to the analysis of field and mesocosm data for determining both causality of and thresholds for measured effects. Some of these have been described by van Dam et al. (2014) and Chariton et al. (2016) and offer promise for being able to derive site‐specific WQBs from complex field data sets (e.g., Threshold Indicator Taxa Analysis [TITAN], Nonlinear canonical analysis of principal coordinates [NCAP]). Recently, some of these tools have been used by Humphrey and Chandler (2018) to 1) infer causation of effects on macroinvertebrate communities associated with mine water discharges and 2) estimate a threshold for the primary contaminant, Mg. Also, Tayler et al. (2018) recently used TITAN to estimate water quality criteria for nutrients based on diatom assemblages in the field.

#### Weight‐of‐evidence process

The limitations associated with historical reliance upon only laboratory toxicity testing have been well recognized. Nowadays, neither the European Commission (2011) nor the USEPA (Cormier et al. 2008) regards any 1 line of evidence as necessarily superior to others in defining a WQB. Moreover, it is now well accepted that stronger inferences can be drawn on issues when multiple lines of evidence are available. Consequently, there has been a recent shift in the favored approach toward the use of multiple lines of evidence, using a WoE process, when deriving site‐specific WQBs (Leung et al. 2014; Merrington et al. 2014; van Dam et al. 2014; USEPA 2016a; Buchwalter et al. 2017; Suter et al. 2017; Chapman 2018). This approach involves the collation and evaluation of information from multiple sources, including data from laboratory and field or semifield studies, and/or mechanistic models, to inform the derivation of a WQB. The USEPA (2016a) recently published formal guidance for using a WoE process in ecological assessment. Although it focuses on using WoE for inferring qualities such as causality of an effect, it also provides guidance on using WoE to derive quantitative results such as WQBs. The guidance was further refined by Suter et al. (2017). Consistent with recent trends internationally, the revised Australian and New Zealand Water Quality Guidelines include a more formal promotion of the use of WoE processes for water quality assessment and the related derivation of site‐specific GVs (ANZG 2018f).

The lines of evidence in a WoE assessment for deriving a site‐specific WQB can include data from different studies (e.g., laboratory, mesocosm, and/or field studies), as well as different and complementary analyses of data from within a study (e.g., those introduced in *Use of field or semifield data*). The evidence may or may not be weighted. The approach to determining a final WQB can involve averaging “candidate” WQBs from across multiple lines of evidence or selecting what is adjudged to be the best estimate of a set of candidate WQBs. Guidance on the benefits and limitations of each approach is provided by USEPA (2016a) and Suter et al. (2017).

This approach is illustrated here by way of an example, with others also available elsewhere (Cormier et al. 2008; Moore et al. 2017; ANZG 2018g; Supervising Scientist 2018). van Dam et al. (2014) described the derivation of site‐specific GVs and establishment of water quality objectives (used in Australia to manage water quality, and largely informed by relevant GVs) for a stream receiving mine discharge water with high electrical conductivity (EC) from the Argyle diamond mine in northern Australia. The lines of evidence included 1) laboratory‐based toxicity testing of the mine waters using locally relevant species in local receiving water; 2) 3 wet seasons of field effects surveys measuring phytoplankton, zooplankton, macroinvertebrate, and fish communities along a gradient of mine water (and EC) contamination at 16 mine‐exposed and 5 to 13 reference sites; and 3) comprehensive water chemistry monitoring and assessment in conjunction with the biological studies. Unfortunately, van Dam et al. (2014) were unable to describe the full details of the entire assessment, much of which remains unpublished in consultancy reports. The WoE assessment was largely qualitative in nature, based on professional best judgment and aided by the fact that strong concordance was observed between the laboratory and field results. The assessment concluded that 1) magnesium sulfate from the mine waters was the most likely cause of measured impacts, 2) effects on the most sensitive species (leptophlebiid mayflies) were measurable above ECs of 250 to 300 µS/cm, and 3) ECs of <300 µS/cm would ensure the maintenance of an intact and functioning aquatic ecosystem despite the loss of some sensitive species.

The effort required in using a WoE process for deriving a site‐specific WQB is usually significant and is unlikely to be required in all cases. However, we believe that it is important to gain as much supporting data and information as possible to improve confidence in a site‐specific WQB to the extent that it is deemed necessary. Typically, the most detailed WOE assessments to derive a site‐specific WQB may be required only where impacts of a contaminant or contaminants are considered a high risk to a high ecological or conservation value ecosystem. Determining the degree of effort required to derive a site‐specific WQB is a key issue and is dealt with in the following section.

## GUIDANCE FOR SELECTING AND ASSESSING APPROACHES

The relative strengths and limitations, and potential applications, of different local WQB derivation approaches outlined in the previous section provide an initial basis upon which to select and/or assess an approach. The current section provides overarching guidance to further assist decision makers with the process.

The nature of the local conditions will often play a significant role in determining the type of derivation approaches that could be used. This consideration relates to the first 2 guiding principles from van Dam et al. (2014) of first understanding the issue, and then adopting an approach that ensures the outcome will address the issue. For example: multiple confounding factors and/or absence of a clear contaminant gradient may limit the value of using a field‐based approach; high temporal variability in key water quality characteristics may limit the usefulness of a single assessment of toxicity of a contaminant to multiple (local or nonlocal) species using local water as the test diluent; or a situation where there are multiple contaminants and/or multiple sources within a localised area may warrant a whole effluent or ambient water toxicity testing approach (also known as direct toxicity assessment) instead, or at least in advance, of deriving a local WQB for a single or several contaminants. Thus, the derivation approach needs to be feasible and meaningful in the context of the local conditions and the broader nature of the issue.

**Figure 3:**
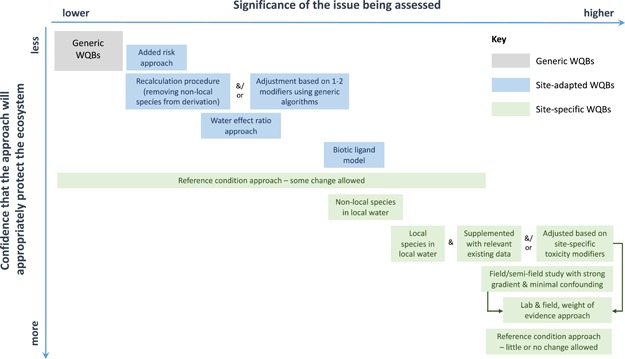
Conceptual hierarchy of approaches for deriving water quality benchmarks (WQBs). Refer to text for descriptions of each method.

Additionally, and as depicted in Figure 3, a relative hierarchy of appropriate WQB derivation approaches, from generic WQBs to WoE‐based site‐specific WQBs, can be conceptualized based on 1) the significance of the issue being assessed and 2) the extent to which the approach provides confidence that the ecosystem in question will be appropriately protected. The positioning of the different WQB approaches within the hierarchy is relative and indicative rather than absolute and quantitative. The significance of the issue being assessed would include, but not necessarily be restricted to, the degree to which one or more of the factors listed in *Why and when we need local water quality benchmarks* was applicable. The extent to which the approach provides confidence that the ecosystem in question will be appropriately protected relates to the certainty that the resulting WQB is neither under‐ nor overprotective for the contaminant and environment in question. Thus, the risk associated with one or more contaminants can be taken into account; for example, where a contaminant will exhibit toxicity at concentrations not much higher than natural background concentrations at the site, greater confidence in the protective concentrations would be required. Consideration of both the significance of the issue and confidence that the ecosystem will be appropriately protected ensures that the concept of fit‐for‐purpose is embedded in the decision‐making process. Consideration of this concept recognizes the need to strike an appropriate balance between the effort to derive a WQB and the risk to an aquatic ecosystem.

Issues associated with lower ecological importance and/or contaminant risk are unlikely to require significant investment in the derivation of site‐specific WQBs based on extensive site‐specific laboratory, field, or WoE studies. Nevertheless, for such situations, there will always be benefit in exploring the extent to which a generic WQB can be adapted to be more relevant to the site of interest, or site‐specific WQBs based on reference site data can be derived, as recommended by the ANZG (2018e). Examples of site‐adapted WQBs are more difficult to find in the published literature, as they typically involve relatively simple calculations with little or no new data generation. As site‐adapted WQBs are less tailored to the site/issue of interest, they should not be used in isolation for significant ecosystems and/or contaminant risks. A possible exception to this might be site‐adapted WQBs for metals derived from well‐developed and validated BLM or MLR models. Otherwise, the derivation and application of site‐adapted WQBs will be more appropriate for less ecologically important ecosystems and/or contaminant risks.

In contrast, where contaminants have the potential to impact a high ecological value and/or sociopolitically sensitive area (e.g., World Heritage areas such as the Great Barrier Reef or Kakadu National Park in Australia, or Wood Buffalo National Park in Canada) and/or their bioavailability and toxicity are likely to be higher than would be the case under typical standard laboratory toxicity testing conditions, then considerable investment to derive rigorous site‐specific WQBs is likely to be required. Ideally, the work program would involve a WoE approach consisting of both laboratory and (semi)field studies (e.g., see Supervising Scientist 2018). Where data from high‐quality field studies are available (see *Use of field or semifield data*), they should carry significantly more weight for deriving site‐specific WQBs than any associated laboratory studies because they fully represent what is occurring in the exposed ecosystem. Thus, wherever possible, existing field data should be assessed for their suitability for deriving site‐specific WQBs (i.e., field data for which confounding is low or can be resolved and a concentration–response relationship is observed for communities of potentially sensitive organisms).

The use of effects‐based approaches assumes that there is acceptance for contaminant concentrations to exceed natural background concentrations but remain below biological effect thresholds. Such assumptions would need to be tested with all key stakeholders. If there was no such acceptance, then the most protective and, in fact, only option is to use the reference condition approach with a management goal of no change relative to the reference condition water quality. If water quality is well below (i.e., poorer than) the reference condition, subsequent attainment of this condition might require an incremental approach to water quality improvement, with interim targets set over defined time stages. In Australia and New Zealand, this approach is consistent with the principle of continual improvement that underpins the approach to water quality management (ANZG 2018a).

Many water quality issues will lie in the middle of each of the 2 axes in Figure 3, with less clarity about decisions to derive site‐adapted or site‐specific WQBs. Where the appropriate approach is not immediately evident, the significance of the issue and necessary level of confidence that the WQB is appropriately protective will need to be further interrogated. Examples of situations in which greater effort may be invested in WQB derivation include the following:

- where community concerns about an issue are strong enough to warrant a higher confidence outcome and, therefore, the derivation of site‐specific WQBs, or even a WoE approach where both site‐adapted and site‐specific WQBs are derived and compared;
- assessments aimed at determining the need or otherwise for large‐scale remediation works, where the development of site‐specific WQBs with associated high confidence may be required; and
- an operator or proponent may be sufficiently motivated to ensure that the approach to deriving local WQBs is as comprehensive and rigorous as possible, to maximize confidence in the outcomes and minimize any regulatory uncertainty. This was the case for the Argyle mine example summarized in *Weight‐of‐evidence process*.
  Alternatively, examples of situations in which less effort may be invested include the following:
- where the area of potential impact is so localized and/or represents only a very small proportion of a particular ecosystem or habitat, then only site‐adapted WQBs may be deemed necessary; and
- where there is a biological monitoring program in place to assess change and from which the data can, over time, be used to refine or even derive a site‐specific WQB, then a site‐adapted WQB may be deemed adequate in the first instance.

The conceptual framework presented here represents a starting point that could be improved upon in the future. It can be used prospectively or retrospectively, that is, at the outset, to determine what might be required to derive a local WQB (e.g., by a proponent or operator), or at the end, to evaluate the appropriateness of an already derived local WQB (e.g., by a regulator) for a given issue. Although there is some appeal in seeking to build upon the framework with a quantitative method for selecting or evaluating approaches for deriving local WQBs, it is highly unlikely that such a tool could incorporate every possible variation in methods for deriving local WQBs and, so, would not add further value at this stage.

### Additional factors

In addition to the guidance provided throughout this review, it is always important to consider factors such as quality control/quality assurance and transparency when assessing the rigor of a local WQB. This assessment should extend to the appropriateness of aspects such as experimental design, sample size, endpoint selection, testing and chemical analysis procedures, statistical analysis of toxicity and effects data, and statistical method for deriving the WQB. Transparency around decision making, especially when outside of standard methods, is critical to the defensibility of a local WQB.

A final requirement for ensuring that a local WQB is rigorous and fit‐for‐purpose is that of independent peer review. Peer review provides an additional layer of technical scrutiny that will ultimately strengthen the defensibility of a WQB and, in doing so, increase the likelihood of it being accepted by regulators. Accordingly, Warne et al. (2018) strongly recommend the peer review of local WQBs.

## SUMMARY

There is no “one size fits all” approach for deriving local WQBs. However, there has been limited guidance available for practitioners deriving and/or assessing WQBs to help them determine which approaches are appropriate for which situations. We have built on the limited existing guidance to hopefully redress this deficiency.

The guidance on derivation and assessment of local WQBs, together with the guiding principles proposed by van Dam et al. (2014) and reproduced in Figure 2, need to be reemphasized. In particular, the need to strike an adequate balance between prescription and flexibility, and to maximize quality and rigor through the appropriate use of the best available science. In addition, the present paper has identified an additional principle, which is to recognize the concept of fit‐for‐purpose. That is, the level of effort and complexity required to derive a local WQB should be commensurate with the significance of the issue, in terms of 1) the importance of the ecosystem and 2) the risk posed by the contaminants. The present paper has outlined key derivation approaches and their relative strengths and limitations, provided example applications, and proposed a conceptual hierarchy of approaches, which can be used prospectively or retrospectively, for determining the requirements for deriving WQBs. We anticipate that the guidance will be of value to industry and regulators alike and, through such education, will result in better environmental management and outcomes.

## Data Accessibility

No data were generated from this review.
